# Genetic Diversity of Spike, 3a, 3b and E Genes of Infectious Bronchitis Viruses and Emergence of New Recombinants in Korea

**DOI:** 10.3390/v5020550

**Published:** 2013-01-31

**Authors:** Mei-Lan Mo, Seung-Min Hong, Hyuk-Joon Kwon, Il-Hwan Kim, Chang-Seon Song, Jae-Hong Kim

**Affiliations:** 1 Laboratory of Avian Diseases, College of Veterinary Medicine, Seoul National University, Seoul 151-742, Korea; E-Mails: momeilan@163.com (M.-L.M.); maldalija@daum.net (S.-M.H.); ilhwan98@snu.ac.kr (I.-H.K.); 2 Research Institute for Veterinary Science , College of Veterinary Medicine, Seoul National University, Seoul 151-742, Korea; E-Mail: kwonhj01@snu.ac.kr; 3 BK21 for Veterinary Science, Seoul National University, Seoul 151-742, Korea; 4 College of Animal Science and Technology, Guangxi University, 100 Daxue Road, Nanning, Guangxi 530005, China; 5 College of Veterinary Medicine, Konkuk University, 1 Hwayang-dong, Gwangjin-gu, Seoul 143-701, Korea; E-Mail: songcs@konkuk.ac.kr

**Keywords:** infectious bronchitis virus (IBV), phylogenetic analysis, genetic diversity, recombinant, vaccine

## Abstract

The nucleotide sequences of a region including S1, S2, 3a, 3b and E genes of twenty-seven infectious bronchitis virus (IBV) isolates in Korea between 1990–2011 were determined and phylogenetic and computational recombination analyses were conducted. The sizes of coding regions of some genes varied among IBV isolates due to deletion or insertion of nucleotides; the nucleotide similarities of S1, S2, 3a, 3b and E genes among the 27 isolates were 75.9%–100.0%, 85%–100.0%, 64.0%–100.0%, 60.4%–100.0% and 83.1%–100.0%, respectively. According to phylogenetic analysis of S1 gene, the 27 isolates were divided into five genotypes, Mass, Korean-I (K-I), QX-like, KM91-like and New cluster 1. The phylogenetic trees based on the S2, 3a, 3b, E genes and S1-S2-3a-3b-E (S1-E) region nucleotide sequences did not closely follow the clustering based on the S1 sequence. The New cluster 1 prevalent during 2009 and 2010 was not found in 2011 but QX-like viruses became prevalent in 2011. The recombination analysis revealed two new S gene recombinants, 11036 and 11052 which might have been derived from recombinations between the New cluster 1 and QX-like viruses and between the K-I and H120 (vaccine) viruses, respectively. In conclusion, multiple IBV genotypes have co-circulated; QX-like viruses have recurred and new recombinants have emerged in Korea. This has enriched molecular epidemiology information of IBV and is useful for the control of IB in Korea.

## 1. Introduction

Infectious bronchitis virus (IBV), a member of genus *Gammacoronavirus*, subfamily *Coronavirinae*, family *Coronaviridae*, order *Nidovirales* [1] is the causative agent of infectious bronchitis (IB), an acute, highly infectious and contagious disease of chickens worldwide. IBV infects the respiratory tract, kidneys and oviduct, resulting in reduced performance, reduced egg quality and quantity, as well as increased susceptibility to infection with other pathogens [2]. Different serotypes of IBVs confer little or no cross-protection against each other. The increasing number of new serotypes of IBV, which were caused by frequent gene mutation and recombination, are a major challenge for the prevention and control of IB [3,4,5]. 

IBV genome consists of a linear, single-stranded, positive-sense RNA of 27.6 kb [6]. Approximately two-thirds of the genome encodes two polyproteins 1a and 1b and the remaining one-third, structural proteins and small nonstructural accessory proteins [7]. IBV encodes four essential structural proteins, the spike (S) glycoprotein, the membrane (M) glycoprotein, the nucleocapsid (N) protein and the envelope or small membrane (E) protein [8]. The S protein post-translationally cleaves into S1 and S2 subunits. The S1 glycoprotein is most variable and contains hypervariable regions carrying epitopes for serotype-specific, virus-neutralizing and hemagglutination-inhibiting antibodies. The S2 glycoprotein contains two antigenic determinants and may affect the S1 specific antibody binding [9,10]. Interactions of E and M proteins are important for virus budding and formation of virus-like particles, which are involved in mucosal immunity [11]. Nonstructural protein genes 3 and 5 intersperse among the structural protein genes [6]. Gene 3 is functionally tricistronic with three open reading frames (ORFs), 3a, 3b, and 3c. Small nonstructural proteins 3a and 3b are encoded by 3a and 3b ORFs, respectively, and structural protein E by ORF 3c [8]. The proteins of ORFs 3a and 3b of IBV are not essential for replication [12].

IBV was first described in 1980 in Korea [13]. Vaccination programs based on live attenuated vaccine H120 strain and inactivated oil-emulsion vaccine containing KM91 and M41 strains have been implemented for many years to control IB [14]. In spite of extensive vaccination, IB is still epidemic in Korea due to the continual emergence of variants [15]. Therefore, it is very crucial to understand the genetic characteristics of Korean IBV field isolates for the control of IB. Some studies on the molecular epidemiology of Korean IBVs have been reported [14,16,17,18]. The studies on Korean IBVs showed that besides K-Ι (respiratory strains), K-ΙΙ (nephropathogenic strains, including KM91-like and QX-like subgroups), and K-ΙΙΙ (enteric strains), some recent isolates formed two different clusters (New cluster 1 and 2) [15]. However, all these studies were merely focused on S1 or even partial S1 gene sequence, therefore, the comprehensive genetic information of circulating IBV strains in Korea available is limited. Genetic analysis on multiple genes sequence has yet to be fully investigated.

Recombination can cause emergence and evolution of different IBV genotypes as well as different species of coronavirus [19]. The studies on whether IBV recombination occurred, the hot-spot for recombination and the effect of recombination on the antigenicity and pathogenicity of IBV are very important, because they can lead to correct forecasting of IBV evolution and thus enable the development of better control methods. More and more recombination events have been reported on IBV and were found to be distributed throughout the entire genome [20,21,22]. Recombination between nephropathogenic KM91 and the QXIBV strain has been documented in Korea based on S1 gene analysis [15,18]. Examining only a small part of the genome may result in misleading conclusions because of point mutations or conserved regions of the gene [23]. Therefore, it is necessary to examine the large part of the genome.

The objective of the present study was to decipher the genetic features of S1, S2, 3a, 3b and E genes of 27 IBVs circulating in Korea and identify recombinants, providing IBV molecular epidemiology information and laying a good foundation for the control of IB in Korea.

## 2. Results

### 2.1. Isolation and Identification of Field Isolates

Twenty seven samples were found to induce the dwarﬁng, stunting, or curling of embryos after three to five blind passages. The allantoic fluids of all of these samples were subjected to RNA extraction and subsequent RT-PCR detection based on the S1 gene of IBV. All of these isolates were identified to be IBV positive by production of the specific bands (data not shown) confirming the presence of IBVs in the samples.

### 2.2. Sequence Determination and Comparison

**Figure 1:**
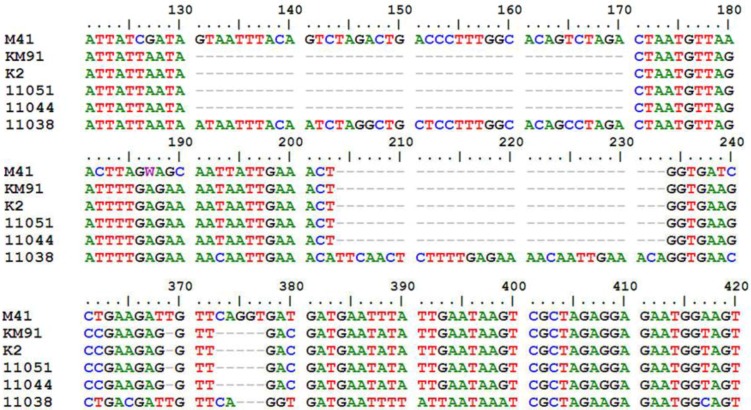
Comparison of 3a and 3b genes of KM91, K2, 11044, 11051 and 11038 strains with M41 strain. Nucleotides of M41 shown start from120th nucleotide after the start codon of 3a gene. Deletion of 40-nt in the 3a genes of KM91, K2, 11044, 11051 strains; Deletion of 5-nt in the 3b genes of KM91, K2, 11044, 11051 strains. Insertion of 30-nt and deletion of 3-nt in the gene 3b of 11038 strain.

ORF sizes of S, 3a, 3b and E gene of the 27 Korean IBVs were compared with those of M41 (accession number GQ504725) in supplementary material (Supplementary Table S1). All of them maintained the classical IBV genome organization of 5'-S-3a-3b-E-3', but the sizes of some corresponding ORFs were variable. The most variable and conserved genes in sizes were S1 and S2, respectively. The S1 ORF comprised 1611 to 1632 nucleotides (nt) and S2 comprised 1878 nt. The ORFs 3b and E contained 189 to 219 nt and 327 to 333 nt, respectively. Compared with the major 3a gene coding 57 amino acids (aa), a 40-nt-deletion was detected at the 3'-end of the 3a genes of KM91, K2, 11044 and 11051, and resulted in a truncated 3a protein with 48 aa (Figure 1). In addition, deletion of 5-nt was found at the 3'-end of the 3b gene in these four IBVs. Insertion of 30-nt and deletion of 3-nt in gene 3b of 11038 resulted in a longer 3b protein with 72 aa (Figure 1).

### 2.3. Alignment Analysis of Nucleotide and Deduced Amino Acid Sequence

Alignment analyses of the S1-E region, S1, S2, 3a, 3b and E genes were performed. The nucleotide and amino acid sequence identities of individual genes among the 27 isolates were 82.1%–100.0%, 75.9%–100.0% (aa: 73.9%–100.0%), 85.2%–100.0% (aa: 87.6%–100.0%), 64.0%–100.0% (aa: 51.7%–100.0%), 60.4%–100.0% (aa: 53.3%–100.0%) and 83.1%–100.0% (aa: 79.2%–100.0%), respectively. The identities of the nucleotide and amino acid sequence of S1-E region, S1, S2, 3a, 3b and E genes between the 27 isolates and all the reference strains were 53.7%–96.5%, 40.0%–99.8% (aa: 17.0%–99.6%), 54.9%–99.4% (aa: 44.7%–98.7%), 63.7%–100% (aa: 50.0%–100%), 69.7%–99.60% (aa: 65.4%–99.0%) and 35.9%–100% (aa: 18.4%–100%), respectively. Among the 27 isolates, 3a and 3b genes shared lower nucleotide and amino acid sequence identities than S1 gene. S2 gene shared the highest nucleotide and amino acid sequences identities. 

Nucleotide and amino acid identities of Korean IBVs to vaccine strains, KM91 and H120 are shown in Supplementary Table S2. Compared with KM91, all other isolates had lower nucleotide homologies (S1: 79.4%–85.3%; S1-E: 83.1%–89.6%; 3a: 64%–79.4%; 3b: 60.4%–82%; E: 85.5%–86%) except for 55.2% (16/27) Korean field isolates sharing the high nucleotide identities (91.4%–99.3% and 90.7%–99.6%) in the S1 gene and S1-E region, K2, 11044 and 11051 sharing 100% in 3a, K2, 11044, 11051 and ES90 isolates sharing 94.7%–100% in 3b, 69% (20/29) isolates sharing 91.5%–99.6% in E genes. Compared with H120, all other isolates had lower nucleotide identities (S1: 77%–83.3%; S1-E: 81.5%–85.4%; 3a: 64%–79.4%; 3b: 60.4%–82%; E: 81.9%–86.3%) except for 11031 (S1, 99.8%; S1-E, 99.8%; 3a, 99.4%; 3b, 100%; E, 99.6%) and 11052 (S1, 92.5%). The majority of Korean isolates showed higher S2 gene nucleotide identities to that of KM91 (93.1%–99.8%) rather than that of H120 (85.1%–87.9%).

Amino acid sequence alignments revealed many point mutations and a few insertions in the S1 gene. The most variations were found between residues 4–26, 53–136, 204–214, 257–300 and 384–400 (H120-numbering) when compared with H120. No amino acid deletion was observed in S1 gene. All or most the Korean isolates showed less amino acid identities than nucleotide identities in S1, 3a, 3b and E genes in contrast to S2 gene, and it indicated that non-synonymous nucleotide changes were selected in them (Supplementary Table S2).

### 2.4. Phylogenetic Analysis

The phylogenetic tree constructed with the Neighbor-joining method and Maximum-likelihood method had very similar topography, so only the Neighbor-joining trees are shown in the present study; the Maximum-likelihood trees are included as supplementary material (Supplementary Figures S1a–f).

**Figure 2:**
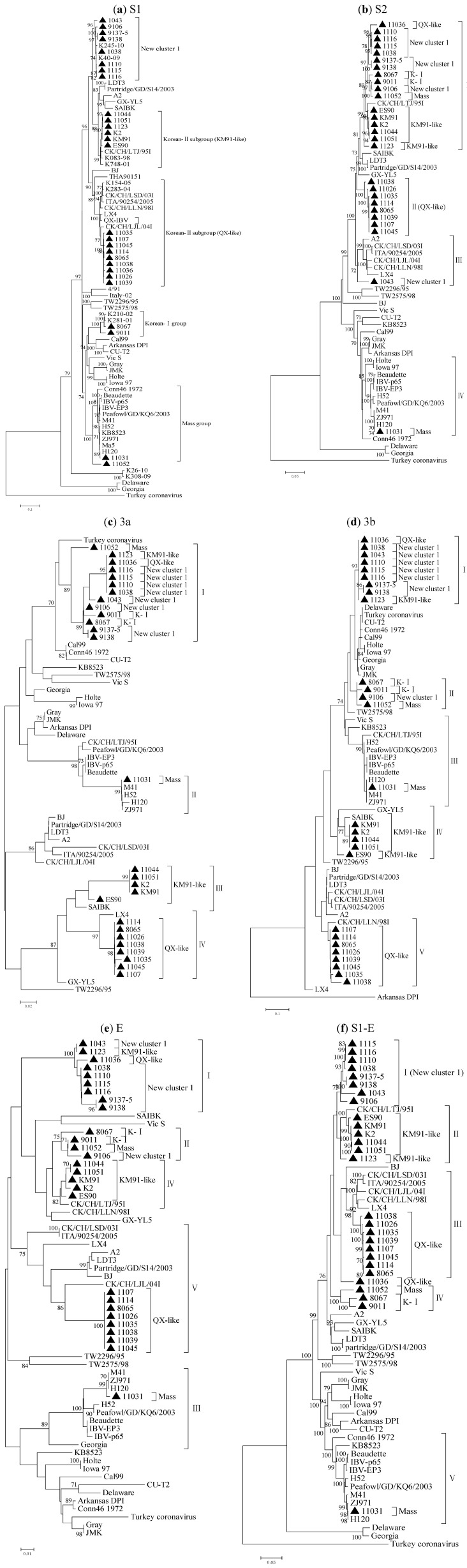
Phylogenetic trees based on nucleotide sequence of S1**(a)**, S2 (**b**), 3a(**c**), 3b (**d**), E (**e**) genes and S1-E region(**f**) of IBVs, where the 27 Korean IBV strains are marked with a filled triangle. Phylogenetic trees were constructed with the neighbor-joining method using MEGA 5.05 version. The bootstrap values were determined from 1000 replicates of the original data. The branch number represents the percentage of times that the branch appeared in the tree. Bootstrap values greater than 50% are shown. The p-distance is indicated by the bar at the bottom of the figure.

Phylogenetic analysis of the S1 gene nucleotide sequences indicated that the 27 Korean isolates were divided into five distinct groups (Figure 2a). Isolates 11031 and 11052 showing close relationship with H120 and M41 were grouped into the Mass group and isolate 11052 occupied a distinct place within this group. Isolates 8067 and 9011 along with the K210-02 and K281-01 strains that were branched into the K-I group previously [18] formed the second group (Korean-Ι group). The third group Korean-ΙΙ subgroup (QX-like) was composed of nine isolates (11035, 1107, 11045, 1114, 8065, 11038, 11036, 1107 and 11039) and eight reference strains including QX-IBV, LX4, CK/CH/LSD/031, ITA/90254/2005, CK/CH/LLN/981, CK/CH/LJL/041 and two Korean strains (K154-05 and K283-04). Six isolates (11044, 11051, 1123, K2, KM91 and ES90) and two published Korean IBV strains (K083-98 and K748-01) and a China IBV strain (CK/CH/LTJ/95I) were classified into the Korean-ΙΙ subgroup (KM91-like). Eight isolates (1043, 9106, 9138, 9137-5, 1038, 1110, 1115 and 1116) along with the Korean strains (K245-10 and K40-09) were categorized into the fifth group (New cluster 1) [15].

The phylogenetic trees based on the S2, 3a, 3b, E genes and S1-E region nucleotide sequences showed that the 27 Korean isolates were segregate into 4, 4, 5, 5 and 5 distinct groups, respectively, which exhibited considerably different topology with the phylogenetic tree of S1 gene (Figure 2b–f). The phylogenetic tree based on S1-E region nucleotide sequences was very similar to that based on S1 gene except for the isolate 11052. The same viruses were grouped together in the two phylogenetic trees based on 3b and E gene sequences. The isolate 11031 was always grouped with H120 and M41 based on the individual genes. Five isolates (11044, 11051, K2, KM91, ES90) of KM91-like group, eight (11035, 1107, 11045, 1114, 8065, 11038, 1107, 11039) of QX-like group and six (9138, 9137-5, 1038, 1110, 1115, 1116) of New cluster 1 group clustered together in the trees based on other genes. Isolates 8067 and 9011 of K-I group clustered together in the trees based on S2, 3b genes and S1-E region. Isolate 11052 (Mass) clustered with K-I group viruses in the trees based on S2, 3b, E genes and S1-E region but with New cluster 1 viruses in the tree based on 3a gene. Isolate 1123 (KM91-like) clustered with New cluster 1 viruses in the trees based on 3a, 3b and E genes. Isolate 11036 (QX-like) clustered with New cluster 1 viruses in the trees based on 3a, 3b and E genes. Isolate 1043 (New cluster 1) did not form a cluster with other isolates in the tree based on S2 gene, but clustered with K-Ι group viruses in the trees based on 3b and E genes. 8067 (K-I) clustered with New cluster 1 viruses in the tree based on 3a gene.

In addition, on combination of the previously published results on IBV in Korea [13,14,15,16,17,18,23] with our result, it was found that QX-like viruses existed in 2008 were not found in 2009, but recurred again in 2010 and showed an increasing tendency in 2011. On the other hand, more viruses of New cluster 1 were isolated in 2009 and 2010. They were dominant viruses in these two years but were not found in 2011 (Supplementary Figure S2).

### 2.5. Analysis of Recombinants

**Figure 3:**
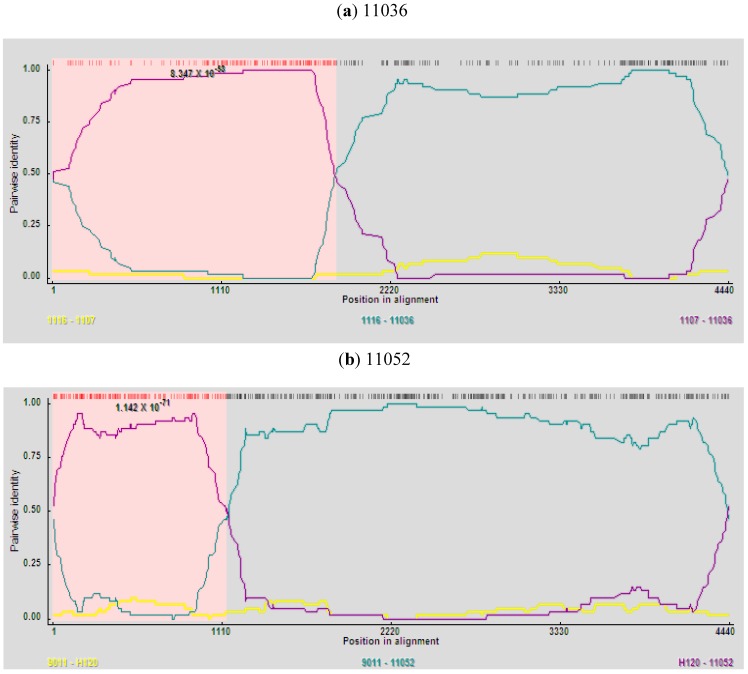
RDP screenshots displaying the possible recombination events on the isolates 11036 and 11052. Each panel displays the pairwise identities among the possible mosaic and its putative parents. Pairwise identity refers to the average pairwise sequence identity within a 60 nt sliding window moved one nucleotide at a time along the alignment of the three sequences. The pink area demarcates the potential recombination regions. **(a)** Comparisons among the putative mosaic 11036 and its putative parents, 1116 and 1107; **(b)** Comparisons among the putative mosaic 11052 and its putative parents, 9011 and H120.

**Figure 4:**
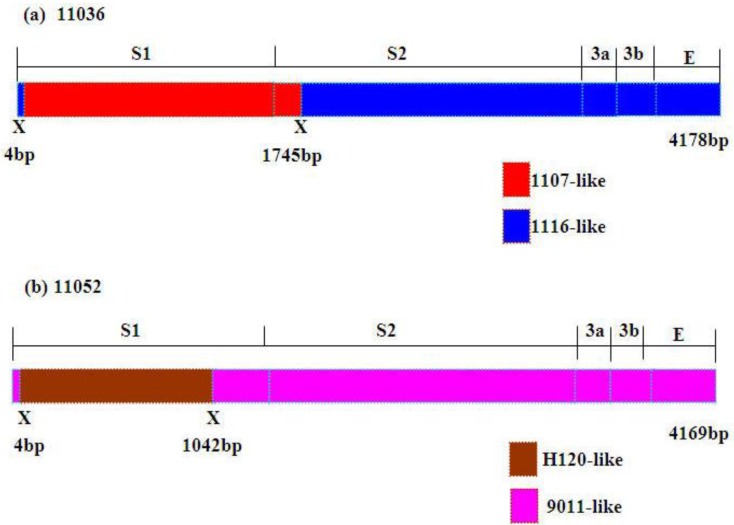
Schematic representation of the S1-S2-3a-3b-E gene region of 11036 and 11052 strains. Putative recombination sitesaredenoted by X.

Among the 27 Korean IBVs examined, recombinant events were detected in the 11036 and 11052 isolates by all recombination detection methods implemented in RDP4.14 software. Isolate 11036 was found to be a mosaic between strains 1116 (New cluster 1) and 1107 (QX-like) (Figure 3a) with very high significance of RDP (8.347 × 10^−58^), GENECONV (8.774 × 10^−43^), BootScan (2.912 × 10^−53^), MaxChi (5.522 × 10^−32^), Chimaera (2.289 × 10^−32^), SiScan (2.587 × 10^−33^ and 3Seq (3.646 × 10^−102^). High significance of RDP (1.147 × 10^−72^), GENECONV (1.219 × 10^−69^), BootScan (1.286 × 10^−66^), MaxChi (2.271 × 10^−29^), Chimaera (4.910 × 10^−30^), SiScan (3.860 × 10^−33^) and 3Seq (8.494 × 10^−110^) also proved that 11052 isolate was a recombinant between strains 9011(K-Ι) and H120 (Figure 3b). Higher similarities supported the potential recombination events of 11036 and 11052. The former part (nt: 4–1745) (nt: 4–1855 in alignment) of S1-E region nucleotide sequence of 11036 showed high similarity with 1107 (99.7%) and the latter part of that with 1116 (99.5%) (Figure 4a). The former part (nt: 4–1042) (nt: 4–1128 in alignment) of S1-E region nucleotide sequence of 11052 showed high similarity with H120 (97.6%) and the remainder region of that with 9011 (98.5%) (Figure 4b). Considerably different topologies were exhibited in the two phylogenetic trees, one constructed from the portion of the alignment between the inferred breakpoints, and the other from the remainder of the alignment, providing further evidence of recombination in 11036 and 11052 (data not shown). The possible recombination regions of the S1-E genes of 11036 and 11052 isolates were confirmed further by Similarity plot and BootScan analyses with the Simplot program (data not shown).

In addition, the recombinant S1 gene was found in all eight New cluster 1 isolates in our study. All the recombinant events of them had the same high significance of RDP (5.980 × 10^−35^), GENECONV (2.954 × 10^−31^), BootScan (3.745 × 10^−34^), MaxChi (2.443 × 10^−16^), Chimaera (2.337 × 10^−16^), SiScan (2.209 × 10^−12^ and 3Seq (2.487 × 10^−35^). The crossover region of six isolates (1043, 9106, 1038, 1110, 1115 and 1116) was observed at nucleotide position 4-907 (4-969 in alignment), and that of two others (9137–5 and 9138) at 66–900 (72–968 in alignment). Their putative parents were proved to be a Korean strain 1123 (KM91-like) and a Chinese strain CK/CH/LSD/03I (QX-like) with higher nucleotide similarities of 95%–97.8% and 97.7%–98.2%, respectively. Significantly discrepant topologies of phylogenetic trees and the results of Similarity plots and BootScan analyses supported further recombinant events of them (data not shown).

## 3. Discussion

The S1 gene is most variable due to scattered hypervariable regions containing epitopes [9,10], but surprisingly our current results indicated that the 3a and 3b genes (64.0%–100.0% and 60.4%–100.0%) were remarkably more variable than S1 gene (75.9%–100.0%). The relatively high variability was due to point mutations and nucleotide deletion or insertion in the coding regions of 3a and 3b. In contrast to S2 gene the amino acid identities were lower than the nucleotide identities in all of the S1 gene and the majority of 3a, 3b and E genes. It reflected that non-synonymous nucleotide changes were selected during virus evolution. The genes with this trait often played important roles in host adaptation and immune response evasion [24]. The roles of S1 and E proteins have been well documented but those of 3a and 3b are still unclear. The replication of recombinant viruses lacking 3a and/or 3b was not affected in chick kidney and in chicken embryos [8,12]. Natural IBV isolates lacking 3a and/or 3b proteins, or expressing truncated 3a protein by a 43 nt-deletion at the 3′-end of the gene have been reported but they replicated normally and showed virulence to chicken embryos and chickens [8,25]. However, the titers of recombinant viruses lacking 3a, decreased earlier than wild type virus after reaching similar titers and a natural IBV isolate lacking start codon of 3a, replicated inefficiently in chicken embryos [8,12]. Most of IBVs maintained intact 3a and 3b genes during evolution and 3a protein localized closely with MxA which plays a role in antiviral activity in smooth endoplasmic reticulum. Therefore, further study to reveal the functions of 3a and 3b are still considered valuable [26]. 

In order to investigate the correlation and parallelism of S1, S2, 3a, 3b, E genes and S1-E region evolution among different viruses, phylogenetic trees based on the individual genes were constructed. According to the previous reports Korean IBV isolates were classified into several genotypes, K-I, K-II, K-III, New cluster 1 and New cluster 2 [14,15]. In this study all genotypes reported and Mass type were identified except K-III and New cluster 2. The result strongly suggested that different genotypes of IBVs have been co-circulating in Korea and genetic bases for new recombinant appearance have been established. The 20 out of 27 isolates showed same clustering patterns in S2, 3a, 3b, E and S1-E region gene-based trees as those in S1 gene-based trees. However, seven isolates (11052, 1123, 11036, 9106, 8067, 9011 and 1043) showed different clustering patterns. The discordance of clustering patterns in S1-based tree and other gene-based trees have already been described by others and may be caused by recombination [23,27,28,29].

A live attenuated vaccine (H120) and an inactivated oil-emulsion vaccine containing KM91 and M41 strains have been used for many years in Korea. A new live attenuated vaccine strain (K2) has been used since 2010 [18]. The protective epitopes are dispersed in the S1 and N-terminal region of S2 genes, and K2 shared almost the same epitopes with KM91. Antigenic variation is related to amino acid change and most of Korean field isolates except KM91-like and Mass type viruses showed more than 10% and 20% amino acid changes compared to KM91 and M41, respectively. This may be the results of virus evolution under vaccine immunity pressure. According to the chronological comparison of each genotype frequency, the K-I group virus has not been isolated since 2009. The New cluster 1 prevalent during 2009 and 2010 was not found in 2011. The finding agreed with the report that K2 vaccine strain used in 2010 could confer cross protection against the new cluster viruses [15]. In contrast QX-like viruses existed in 2008 and disappeared in 2009 but recurred in 2010 with increasing tendency in 2011. These findings may reflect incomplete protection efficacy of commercial vaccines against QX-like viruses, therefore further investigation may be required to clarify the serological diversity of the QX-like and New cluster 1 viruses. The three isolates 1123, 11044 and 11051 of the KM91-like group were isolated from chicken flocks vaccinated with K2. The 11044 and 11051 were isolated within two weeks of vaccination and maintained the genetic traits of K2, 40 nt and 5 nt deletions in 3a and 3b, respectively, as well as high nucleotide identities of analyzed genes. However, 1123 did not show the genetic traits of K2 and formed a separate cluster from other KM91-like viruses in 3a, 3b and E gene-based trees. Thus the pathogenicity of K2-derived field strains should be assessed in further study. 

Recombination can occur between field isolates [5,30] or between field and vaccine viruses [31,32]. In our study, convincing evidence showed that recombination event had occurred in the S1 genes of all eight New cluster 1, 11036 (QX-like) and 11052 (Mass) isolates. The New cluster 1 virus was first identified in 2007 and possessed the former half of the S1 gene from QX-like virus and the latter half from KM91-like virus [15]. In a previous study only S1 gene was analyzed and the origins of other genes were unclear. Our results showed recombination events occurred in the S1 gene of New cluster 1 viruses, with their putative parental strains being Korean strain 1123 (KM91-like) and China strain CK/CH/LSD/03I (QX-like), and two crossover regions (nt: 4–907 and 66–900) were observed. All New cluster 1 viruses except for 9106 strain shared high nucleotide and amino acid identities with 1123 strain in S2, 3a, 3b and E gene, indicating that the latter half of S1, S2, 3a, 3b and E genes originated from the KM91-like virus, 1123. Therefore, most of the New cluster 1 viruses were generated by acquisition of the first 900 nucleotides of QX-like virus S1 gene by a KM91-like virus. However, the fact that the 9106 strain shared relatively low nucleotide and amino acid identities with KM91 strain in the S2 gene, reflects that the New cluster 1 viruses are not clonal. 

The 11036 isolate was a recombinant between 1116 (New cluster 1) and 1107 (QX-like) and this is the first report on the recombination between the genotypes. The 11036 isolate was generated by acquisition of the entire S1 gene and partial S2 gene (amino acid residues 2–581) of 1107 (QX-like group) and remaining S2 gene from 1116 (New cluster 1). Considering the clustering of 11036 with the New cluster 1 viruses in 3a, 3b and E gene-based trees, 11036 may be derived from a New cluster 1 virus having acquired S1 and partial S2 of QX-like virus to evade K2 (KM91)-induced humoral immunity.

The 11052 was a recombinant between the 9011 (K-I) and vaccine strain H120, and it was first report in Korea. Recombination between field virus and H120 has been already reported in other countries but the recombination patterns were different from 11052 [31,32]. The 11052 isolate might be generated by acquisition of the three-quarters (amino acid residues 2–347) of S1 gene from H120 strain (Mass group) and the remaining one-quarter S1 and complete S2 from 9011 (K-I). Considering the clustering of 11052 with the K-I viruses in S2, 3a, 3b and E gene-based trees 11052 may be derived from K-I virus acquiring three-quarters of S1 of H120 to evade K2 (KM91)-induced humoral immunity.

Most of the protective epitopes are located in the first and third quarters of the S1 subunit [9] and two antigenic regions within S2 (between amino acid residues 546–577) were identified as an immune-dominant region [33]. The recombination event occurring in most of the New cluster 1 viruses changed epitopes D, E and partial epitopes C in S1 of a KM91-like virus (1123) with other epitopes maintained. The recombination event occurring in 11036 exchanged all the epitopes of S1 and S2 of a New cluster 1 virus (1116) with those of a QX-like virus (1107), and that in 11052 exchanged epitopes D, E, C, A, B of S1 of a K-I virus (9011) with those of H120. These findings provide valuable information on the importance of these epitopes for humoral immunity evasion and witness the struggling of KM91-like and K-I viruses to survive under immune pressures. However QX-like viruses maintained their own genetic integrity consistently. 

According to our study, 27 isolates were divided into five genotypes, Mass, K-I, QX-like, KM91-like and New cluster 1. Among the 27 isolates, possible recombination event had been predicted in the S1 genes of ten isolates including 11036, 11052 and eight New cluster 1 isolates. Also, the possible recombination events had been predicted between New cluster 1 and QX-like, K-I and vaccine strain H120 (Mass), KM91-like and QX-like, respectively. The predicted recombination events involved all five genotypes. The extensiveness of predicted recombinations in IBV is more than formerly thought. What is more, we found recombinant 11036 might come from recombinant virus (New cluster 1). These results reflected the complex and reticulated evolution of IBV. Hot spots tend to lie immediately upstream of the S glycoprotein gene, as well as in nonstructural proteins 2, 3 and 16, although evidence of recombination was found in every sequence analyzed and was distributed throughout the entire genome [22]. All the hot spots in our study lie immediately upstream of the S glycoprotein gene. The S1 subunit plays a role in attachment to host cell receptors and contains conformationally-dependent virus-neutralizing and serotype-specific epitopes [22]. Spike is also involved in membrane fusion and viral entry into the host cell [22]. The breakpoints near or in spike have the potential to lead to the emergence of new serotypes of IBV or new coronaviruses [22]. The genetic diversity and recombinant events of Korean IBV isolates explains why IBV isolates could escape immune response and obtain adaptability in Korea. 

## 4. Experimental

### 4.1. Virus Isolation and Propagation

Twenty seven Korean IBV field isolates between 1990–2011 (Table 1) were isolated and propagated in 9–11 day-old specific pathogen free embryonated chicken eggs (ECE; VALO BioMedia, Adel, IA, USA) via the allantoic cavity route. Allantoic fluids were harvested at 48 h post-inoculation. Three to 5 blind passages were performed until dwarfing, stunting, or curling of embryos was observed. The viruses were identified by the presence of IBV genome using RT-PCR.

**Table 1:** Korean IBV isolates analyzed in this study.

IBV strain	Year of isolation	Pathogenesis	Type of chicken ^b^	Location ^c^	Vaccination	Accession numbers
ES90	1990	Nephropathogenic	B	Chungcheong	NA	JQ920406
KM91	1991	Nephropathogenic	B	GyeongGi	NA	JQ920377
K2	2001	Attenuated vaccine strain	B	NA	NA	JQ920378
8065	2008	Nephropathogenic, Enteric	L	GyeongGi	Yes	JQ920387
8067	2008	Nephropathogenic	L	GyeongGi	Yes	JQ920388
9011	2009	Respiratory, Nephropathogenic	BB	Chungcheong	NA	JQ920389
9106	2009	Respiratory, Nephropathogenic	L	Chungcheong	Yes	JQ920390
9137-5	2009	Respiratory	B	NA	NA	JQ920391
9138	2009	Enteric	BB	GyeongGi	Yes	JQ920395
1038	2010	NA ^a^	L	GyeongGi	NA	JQ920379
1043	2010	NA	NA	NA	NA	JQ920380
1107	2010	Respiratory	NC	GyeongGi	NA	JQ920381
1110	2010	Respiratory, Nephropathogenic, Enteric	NA	Jeju	Yes	JQ920382
1114	2010	Respiratory	NA	Chungcheong	Yes	JQ920383
1115	2010	Respiratory, Nephropathogenic, Enteric	NA	Jeju	NA	JQ920384
1116	2010	Respiratory, Nephropathogenic, Enteric	NA	Jeju	NA	JQ920385
1123	2010	NA	LB	GyeongGi	Yes	JQ920386
11026	2011	Nephropathogenic	NC	GyeongGi	NA	JQ920396
11031	2011	Respiratory	BB	GyeongGi	NA	JQ920397
11035	2011	Respiratory	L	GyeongGi	Yes	JQ920398
11036	2011	Nephropathogenic	B	Jeolla	NA	JQ920399
11038	2011	Enteric	NC	Chungcheong	NA	JQ920400
11039	2011	Respiratory, Nephropathogenic	B	Jeolla	NA	JQ920401
11044	2011	Respiratory, Nephropathogenic	NA	Gyeongsang	Yes	JQ920402
11045	2011	Respiratory, Nephropathogenic	NA	Jeolla	NA	JQ920403
11051	2011	Respiratory, Nephropathogenic	B	Gyeongsang	Yes	JQ920404
11052	2011	Nephropathogenic	L	Chungcheong	Yes	JQ920405

^a ^NA: data not available. ^b ^B: broiler; L, layer; BB, broiler breeder; NC, native chicken; LB, layer breeder. ^c^ Area where the viruses were isolated

### 4.2. RNA Extraction, RT-PCR, Cloning and Sequencing

Viral RNA was extracted from the infectious allantoic fluid by Viral Gene-spin kit (iNtRON Biotechnology, Seongnam, Korea) according to the manufacturer’s instruction. The RT-PCR reaction was carried out using Onestep RT-PCR kit (Qiagen GmbH, Hilden, Germany) according to the manufacturer’s protocol. Three overlapping fragments were synthesized and amplified with gene specific primers, which were designed in the conserved region of M41 (DQ834384) and described in Table 2. The RT reaction for synthesis of cDNA was incubated at 50 °C for 30 min, and then heated at 95 °C for 15 min to inactivate reverse transcriptase. The PCR was performed with 40 cycles of denaturation at 94 °C for 30 s, anneaning at 50 °C for 30 s, extension at 72 °C for 2 min and final extension at 72 °C for 5 min. The PCR amplicons were separated on 1.5% agarose gel and purified using a PCR purification kit (Macrogen Co. Seoul, Korea) and then sequenced directly or cloned intopGEM®-T Easy Vector (Promega, Madison, WI, USA) following the manufacturer’s instructions and sequenced with ABI3711 automatic sequencer (Macrogen Co. Seoul, Korea). Each region of the fragment was sequenced 2 times from PCR products or 4 to 5 times from independent clones.

**Table 2:** Sequence and position of the primers used in RT-PCR.

Primers	Sense ^a^	Sequence (5′→3′)	Position in genome ^b^	Length	Gene
P1	+	ACAGAGACAAGTTGGCAYGA	20035-20054	1845 bp	Partial of S1
P2	−	CCWGAMACTACAAACTGYTG	21860-21879		
P3	+	AAGAGYGRTGGCTCTCGTA	21580-21598	1800 bp	Partial of S1 and S2
P4	−	GTAACTAYATCTCCTGCAGT	23360-23379		
P5	+	TGCACCTAATGGYATAGTGT	23158-21579	1607 bp	Partial of S2, 3a, 3b, and 3c(E)
P6	+	ACAGAGACAAGTTGGCAYGA	24745-24764		

^a^ Negative-sense (−) or positive-sense (+) primer; ^b^ The nucleotide positions correspond to those in the sequence of the IBV M41 genome (GenBank accession number DQ834384).

### 4.3. ORF Determination and Sequence Analysis

The overlapping sequences of three fragments were assembled together using ChromasPro version 1.5 (Technelysium Pty Ltd., Brisbane, Australia) and ORFs of S, 3a, 3b and E gene were determined using the DNAstar version (DNAStar, Madison, WI, USA). All the entire nucleotide sequences of S-3a-3b-E gene were submitted to the GenBank database and assigned accession numbers listed in Table 1. The nucleotide and the deduced amino acid sequences alignments were generated using the ClustalW Multiple Alignment method of BioEdit version 7.0.9.0 and compared with those of 35 reference IBV strains (Except for S1 gene compared with 50 reference IBV strains) retrieved from the GenBank database with the accession numbers listed in supplementary material (Supplementary Table S3). A comprehensive phylogenetic study based on the nucleotide sequence of individual genes was conducted in order to determine the genetic relationship between different genes. Phylogenetic trees were constructed with the Neighbor-joining method and Maximum-likelihood method using MEGA 5.05 version. Neighbor-joining trees and Maximum-likelihood trees were constructed based on the Kimura-2 parameter model and Tamura-Nei model, respectively. The bootstrap values were determined from 1,000 replicates of the original data.

### 4.4. Computational Recombination Analysis

Aligned nucleotide sequences of S1-E region were analyzed with the Recombination Detection Program (RDP4, Version 4.14) [34] to detect potential recombination events. Increasing the window size was shown to increase the ratio of recombination signals relative to mutational “noise” [22]. Considering IBV has a high mutation rate, which can mask recombination signals, the window size was increased from the default setting 30 bp to 60 bp. The highest acceptable P value was 0.05 and the detection of recombination events was applied between sequences sharing 0 and 100% identity. Seven algorithms in RDP 4.14, including RDP, GENECONV, BootScan, MaxChi, Chimaera, SiScan and 3Seq were used to evaluate the recombination events. Two phylogenetic trees, one constructed from the portion of the alignment between the inferred breakpoints, and the other from the remainder of the alignment were drawn and compared to assess recombination events further. Similarity plot and BootScan analyses were performed using the SimPlot program (version 3.5.1) to confirm further recombination events and recombination breakpoints.

## 5. Conclusions

In conclusion, multiple IBV genotypes have co-circulated; QX-like viruses have re-occurred and new recombinants have emerged in Korea. This has enriched molecular epidemiology information of IBV and is useful for the control of IB in Korea.

## Supplementary Files

Supplementary File 1 Supplementary Material (PDF, 717 KB)
