# Supplementary material for: Genetic Diversity of Spike, 3a, 3b and E Genes of Infectious Bronchitis Viruses and Emergence of New Recombinants in Korea
Source: Viruses. 2013 Jan 31;5(2):550–67. doi: 10.3390/v5020550 (PMC3640513; doi:10.3390/v5020550)
Supplement: Supplementary File 1 — Supplementary Material (PDF, 717 KB) [file viruses-05-00550-s001.pdf]

Supplementary Material

Figure S1. Phylogenetic trees based on nucleotide sequence of S1 (**a**), S2 (**b**), 3a (**c**), 3b (**d**), E (**e**) and S1-E (**f**) genes of IBVs, where the 27 Korean IBV strains are marked with a filled triangle. Phylogenetic trees were constructed with the Maximum-likelihood method using MEGA 5.05 version. The bootstrap values were determined from 1000 replicates of the original data. The branch number represents the percentage of times that the branch appeared in the tree. Bootstrap values greater than 50% are shown. The p-distance is indicated by the bar at the bottom of the figure.

(**a**) **S1**

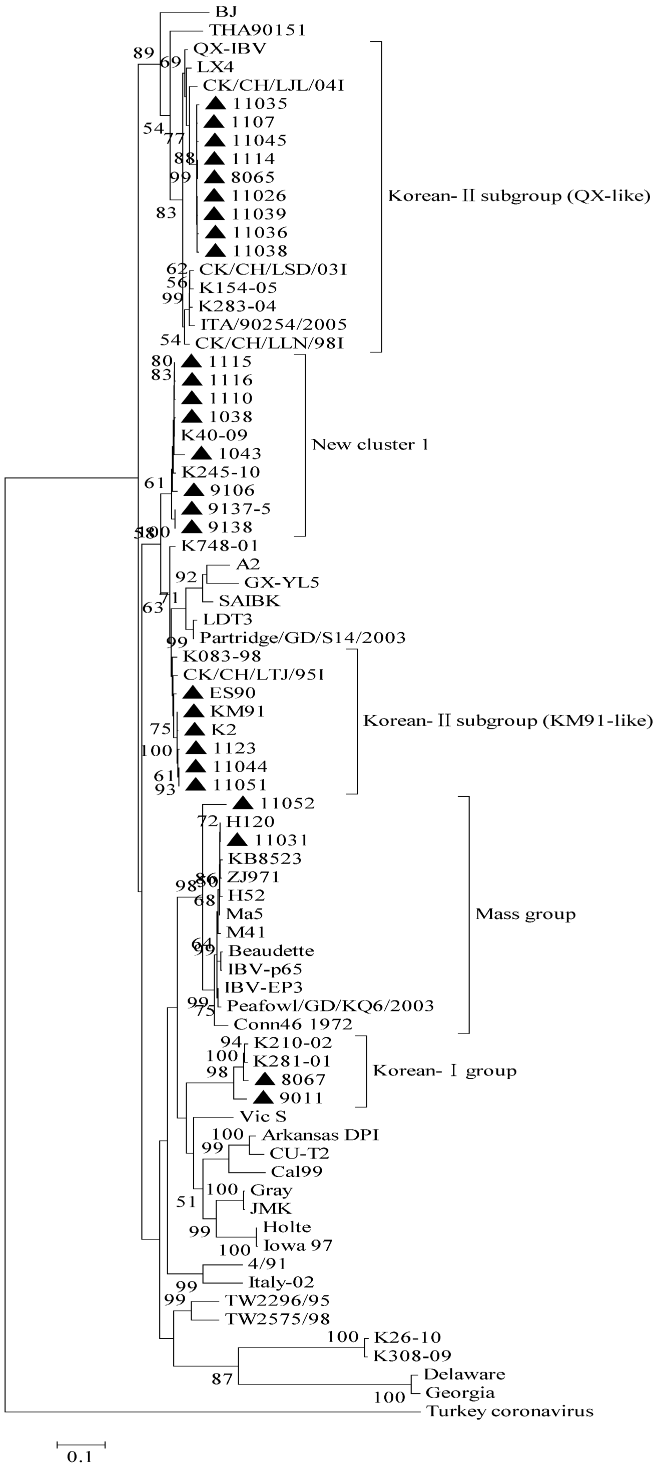

(b) S2

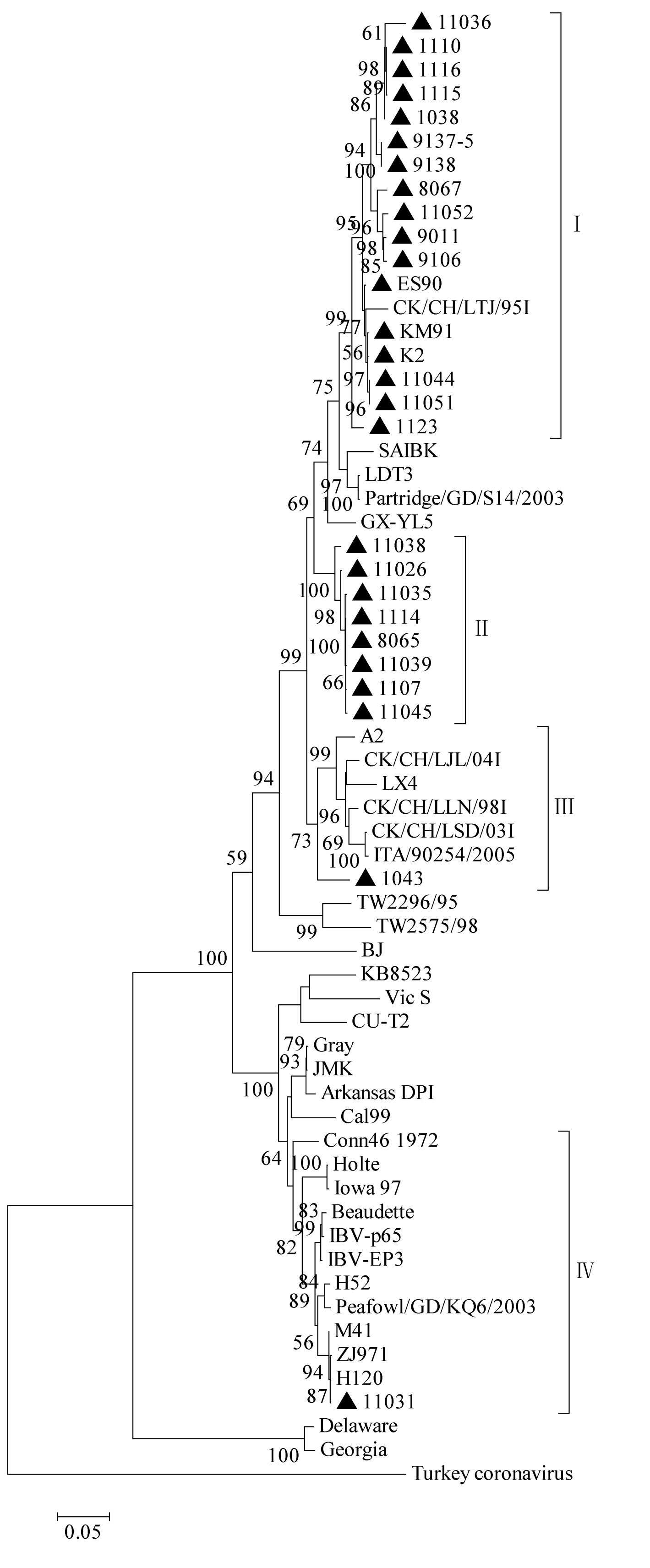
11036
1110
1116
1115
1038
9137-5
9138
8067
11052
9011
9106
ES90
CK/CH/LTJ/95I
KM91
K2
11044
11051
1123
I
SAIBK
LDT3
Partridge/GD/S14/2003
GX-YL5
11038
11026
11035
1114
8065
11039
1107
11045
II
A2
CK/CH/LJL/04I
LX4
CK/CH/LLN/98I
CK/CH/LSD/03I
ITA/90254/2005
1043
III
TW2296/95
TW2575/98
BJ
KB8523
Vic S
CU-T2
Gray
JMK
Arkansas DPI
Cal99
Conn46 1972
Holte
Iowa 97
Beaudette
IBV-p65
IBV-EP3
H52
Peafowl/GD/KQ6/2003
M41
ZJ971
H120
11031
IV
Delaware
Georgia
Turkey coronavirus
0.05

(c) 3a

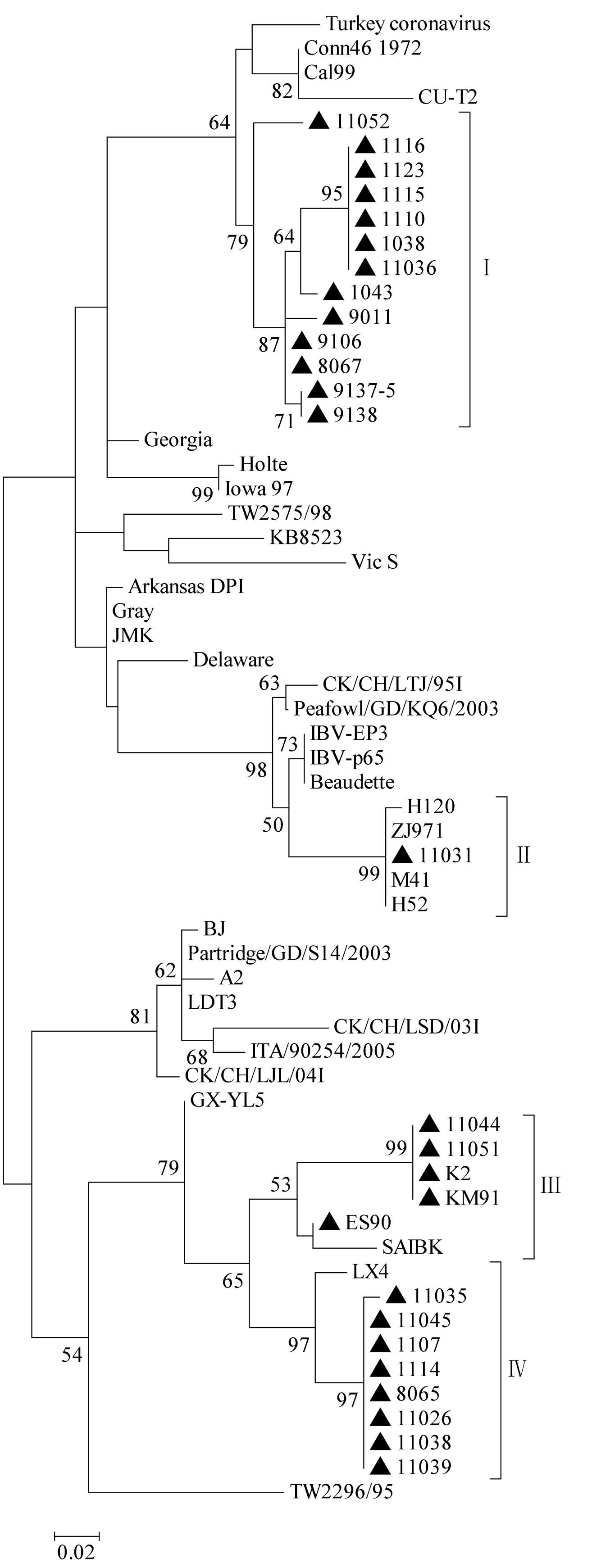
Turkey coronavirus
Conn46 1972
Cal99
CU-T2
11052
1116
1123
1115
1110
1038
11036
1043
9011
9106
8067
9137-5
9138
I
Georgia
Holte
Iowa 97
TW2575/98
KB8523
Vic S
Arkansas DPI
Gray
JMK
Delaware
CK/CH/LTJ/95I
Peafowl/GD/KQ6/2003
IBV-EP3
IBV-p65
Beaudette
H120
ZJ971
11031
M41
H52
II
BJ
Partridge/GD/S14/2003
A2
LDT3
CK/CH/LSD/03I
ITA/90254/2005
CK/CH/LJL/04I
GX-YL5
11044
11051
K2
KM91
ES90
SAIBK
III
LX4
11035
11045
1107
1114
8065
11026
11038
11039
IV
TW2296/95
0.02

(d) 3b

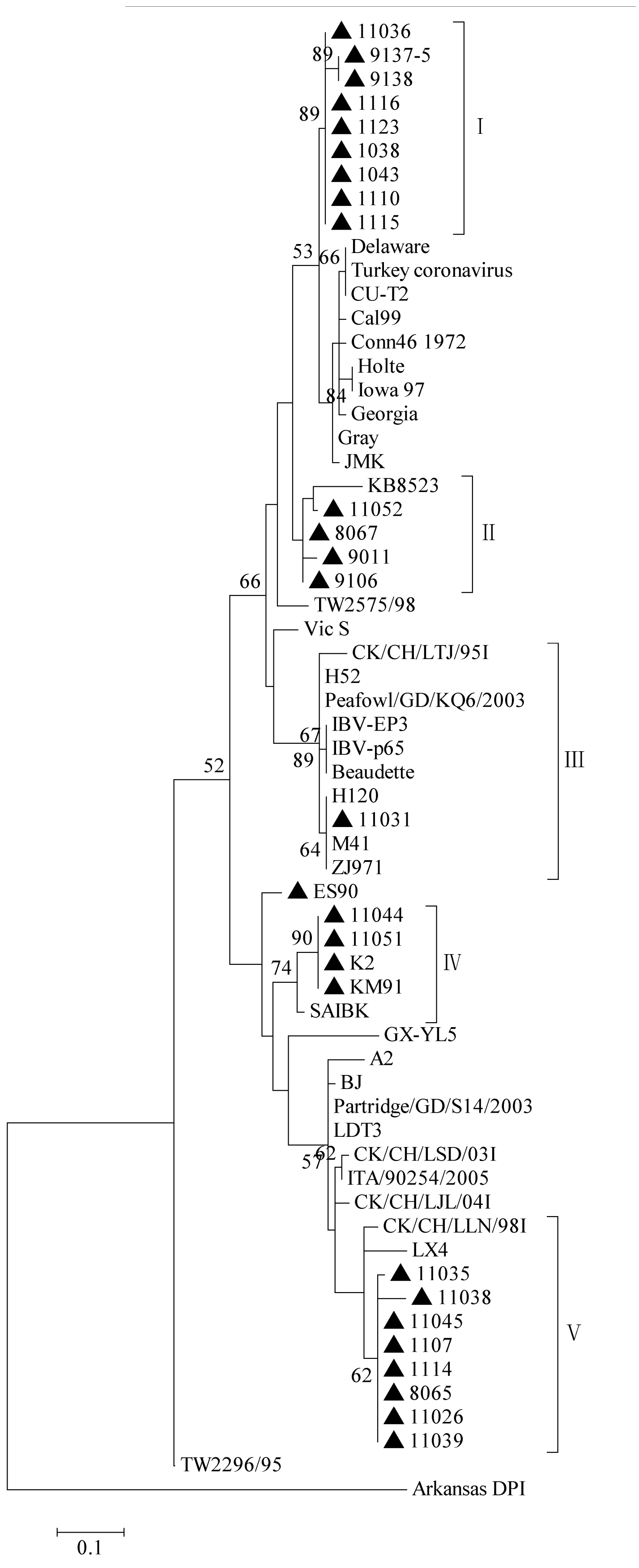
11036
9137-5
9138
1116
1123
1038
1043
1110
1115
I
Delaware
Turkey coronavirus
CU-T2
Cal99
Conn46 1972
Holte
Iowa 97
Georgia
Gray
JMK
KB8523
11052
8067
9011
9106
II
TW2575/98
Vic S
CK/CH/LTJ/95I
H52
Peafowl/GD/KQ6/2003
IBV-EP3
IBV-p65
Beaudette
H120
11031
M41
ZJ971
III
ES90
11044
11051
K2
KM91
SAIBK
IV
GX-YL5
A2
BJ
Partridge/GD/S14/2003
LDT3
CK/CH/LSD/03I
ITA/90254/2005
CK/CH/LJL/04I
CK/CH/LLN/98I
LX4
11035
11038
11045
1107
1114
8065
11026
11039
V
TW2296/95
Arkansas DPI
89
89
53
66
84
66
67
89
64
52
90
74
57
62
62
0.1

(e) E

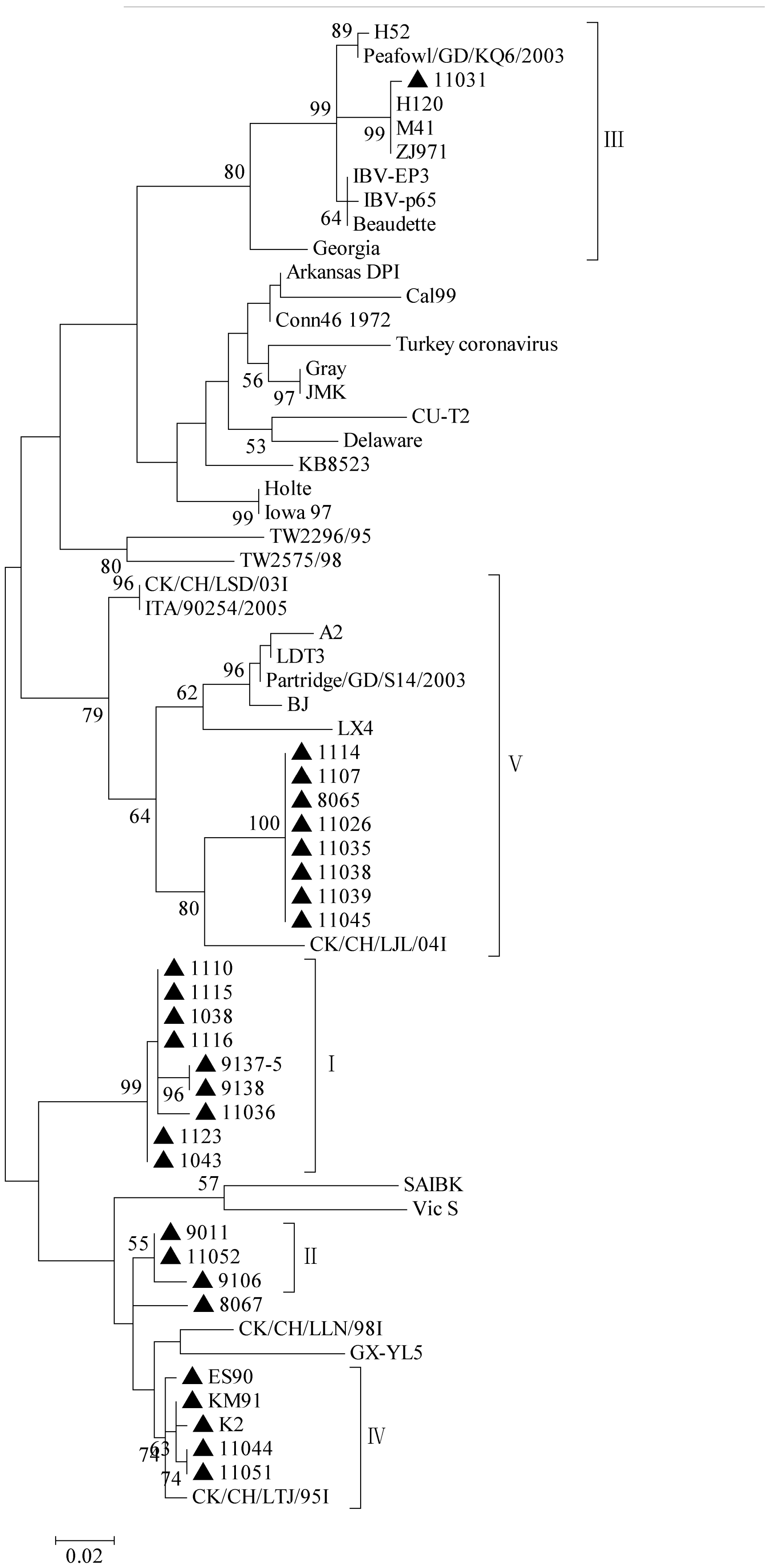
H52
Peafowl/GD/KQ6/2003
11031
H120
M41
ZJ971
IBV-EP3
IBV-p65
Beaudette
Georgia
Arkansas DPI
Cal99
Conn46 1972
Turkey coronavirus
Gray
JMK
CU-T2
Delaware
KB8523
Holte
Iowa 97
TW2296/95
TW2575/98
CK/CH/LSD/03I
ITA/90254/2005
A2
LDT3
Partridge/GD/S14/2003
BJ
LX4
1114
1107
8065
11026
11035
11038
11039
11045
CK/CH/LJL/04I
1110
1115
1038
1116
9137-5
9138
11036
1123
1043
SAIBK
Vic S
9011
11052
9106
8067
CK/CH/LLN/98I
GX-YL5
ES90
KM91
K2
11044
11051
CK/CH/LTJ/95I
III
V
I
II
IV
0.02

(f) S1-E

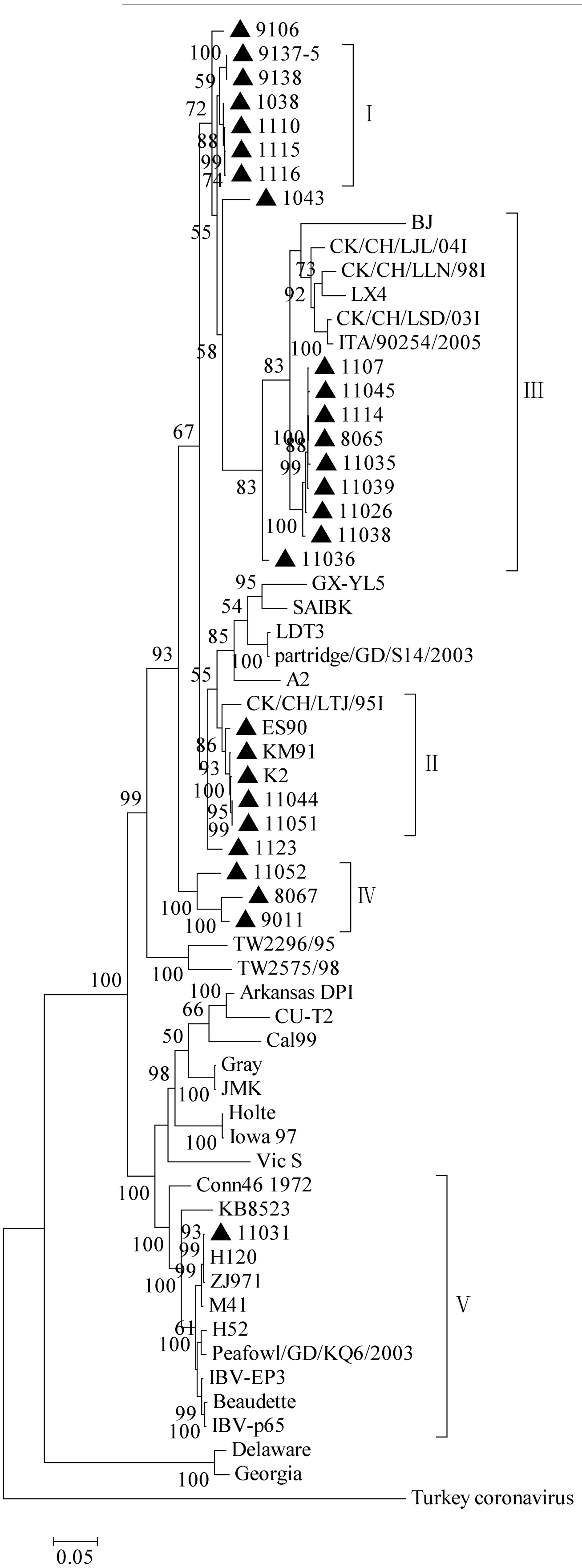
9106
9137-5
9138
1038
1110
1115
1116
1043
I
BJ
CK/CH/LJL/04I
CK/CH/LLN/98I
LX4
CK/CH/LSD/03I
ITA/90254/2005
1107
11045
1114
8065
11035
11039
11026
11038
11036
III
GX-YL5
SAIBK
LDT3
partridge/GD/S14/2003
A2
CK/CH/LTJ/95I
ES90
KM91
K2
11044
11051
1123
II
11052
8067
9011
IV
TW2296/95
TW2575/98
Arkansas DPI
CU-T2
Cal99
Gray
JMK
Holte
Iowa 97
Vic S
Conn46 1972
KB8523
11031
H120
ZJ971
M41
H52
Peafowl/GD/KQ6/2003
IBV-EP3
Beaudette
IBV-p65
V
Delaware
Georgia
Turkey coronavirus
0.05

Figure S2. Virus numbers of genotypes isolated during different years.

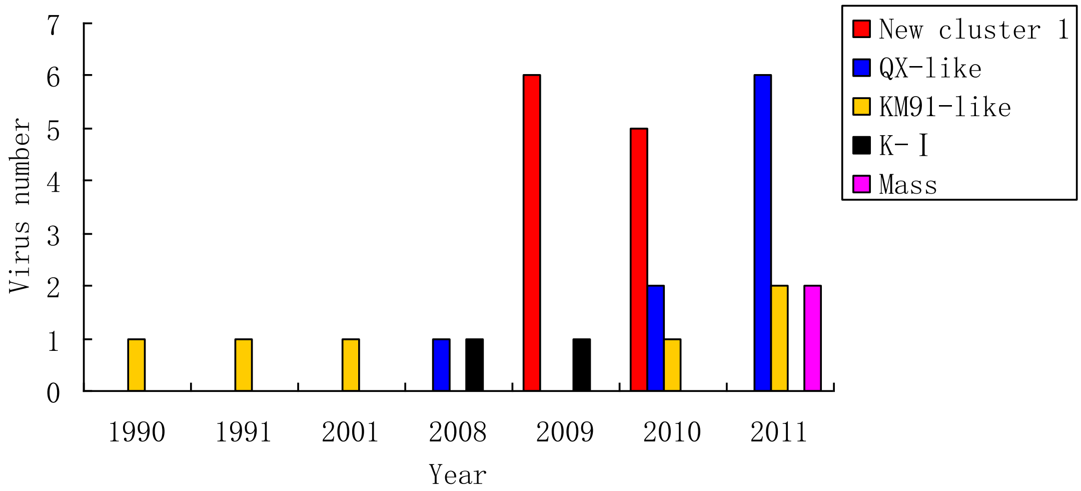

Table S1. Comparison of ORF sizes in the S, 3a, 3b and E gene encoding regions of IBV [a].

Strains	Genotype	S1	S2	Gene 3				
				3a	Del (nt)	3b	Del/ins (nt)	3c/E
M41	Mass	1611(537)	1878(625)	174（57）	-	195(64)	-/-	330(109)
11031	Mass	1611(537)	1878(625)	174（57）	-	195(64)	-/-	330(109)
11052	Mass	1611(537)	1878(625)	174（57）	-	195(64)	-/-	333(110)
8067	Korean type I	1632(544)	1878(625)	174（57）	-	195(64)	-/-	327(108)
9011	Korean type I	1629(543)	1878(625)	174（57）	-	195(64)	-/-	333(110)
8065	QX-like	1620(540)	1878(625)	174（57）	-	189(62)	5/-	327(108)
1107	QX-like	1614(538)	1878(625)	174（57）	-	189(62)	5/-	327(108)
1114	QX-like	1620(540)	1878(625)	174（57）	-	189(62)	5/-	327(108)
11026	QX-like	1617(539)	1878(625)	174（57）	-	189(62)	5/-	327(108)
11035	QX-like	1620(540)	1878(625)	174（57）	-	189(62)	5/-	327(108)
11036	QX-like	1620(540)	1878(625)	174（57）	-	195(64)	-/-	333(110)
11038	QX-like	1620(540)	1878(625)	174（57）	-	219(72)	3/30	327(108)
11039	QX-like	1620(540)	1878(625)	174（57）	-	189(62)	5/-	327(108)
11045	QX-like	1620(540)	1878(625)	174（57）	-	189(62)	5/-	327(108)
ES90	KM91-like	1620(540)	1878(625)	174（57）	-	189(62)	5/-	330(109)
KM91	KM91-like	1614(538)	1878(625)	147（48）	40	189(62)	5/-	330(109)
K2	KM91-like	1611(537)	1878(625)	147（48）	40	189(62)	5/-	330(109)
1123	KM91-like	1617(539)	1878(625)	174（57）	-	195(64)	-/-	333(110)
11044	KM91-like	1611(537)	1878(625)	147（48）	40	189(62)	5/-	330(109)
11051	KM91-like	1611(537)	1878(625)	147（48）	40	189(62)	5/-	330(109)
9106	New cluster-I	1620(540)	1878(625)	174（57）	-	195(64)	-/-	333(110)
9137-5	New cluster-I	1614(538)	1878(625)	174（57）	-	195(64)	-/-	333(110)
9138	New cluster-I	1614(538)	1878(625)	174（57）	-	195(64)	-/-	333(110)
1038	New cluster-I	1620(540)	1878(625)	174（57）	-	195(64)	-/-	333(110)
1043	New cluster-I	1620(540)	1878(625)	174（57）	-	195(64)	-/-	333(110)
1110	New cluster-I	1620(540)	1878(625)	174（57）	-	195(64)	-/-	333(110)
1115	New cluster-I	1620(540)	1878(625)	174（57）	-	195(64)	-/-	333(110)
1116	New cluster-I	1620(540)	1878(625)	174（57）	-	195(64)	-/-	333(110)

[a] The sequence of the M41 genome was used as reference, GenBank accession no. GQ504725. The sizes of amino acids are indicated in parentheses.

Table S2. Pair-wise comparison of nucleotide and deduced amino acid sequences of Korean IBVs with vaccine strains KM91 and H120.

Strains	Nucleotide and amino acid sequence identities (%) [a]											
	S1-E		S1		S2		3a		3b		E	
	KM91	H120	KM91	H120	KM91	H120	KM91	H120	KM91	H120	KM91	H120
11031	83.1	**99.8**	82.4(81.4)	**99.8(99.6)**	*85.5(88)*	***99.8(99.8)***	65.7(51.7)	**99.4(98.2)**	82(76.9)	**100(100)**	85.5(83.7)	**99.6(99)**
11052	89.3	87.6	82(78.8)	**92.5(90.5)**	***97.5(97.7)***	*85.4(87.6)*	65.7(51.7)	82.1(79.3)	81(70.7)	86.6(72.3)	***96.3(98.1)***	84.9(84.6)
8067	87.9	82.6	79.4(77)	78.7(76.8)	**97.3(97.1)**	*85.4(87.5)*	65.1(51.7)	81.6(79.3)	80.5(72.3)	86.1(73.8)	**95.1(94.5)**	81.9(79.2)
9011	88.4	82.6	79.8(76.6)	78.5(76.8)	**97.4(97.2)**	*85.5(87.6)*	64(53.4)	80.4(77.5)	80.5(72.3)	86.1(70.7)	***97.2(98.1)***	*84.6(84.6)*
8065	88.2	82.1	85.3(85.1)	77(76.1)	***93.6(95.5)***	*86.2(89.1)*	74.8(68.9)	82.7(70.6)	71.7(64.6)	76.9(63)	86(85.4)	86(82.7)
1107	88.2	82.1	85.1(85)	77(75.9)	***93.6(95.3)***	*86.1(88.9)*	74.8(68.9)	82.7(70.6)	71.7(64.6)	76.9(63)	86(85.4)	86(82.7)
1114	88.2	82.1	85.3(85.1)	77(76.1)	***93.6(95.5)***	*86.2(89.1)*	74.8(68.9)	82.7(70.6)	71.7(64.6)	76.9(63)	86(85.4)	86(82.7)
11026	88.3	82.1	85.4(85.3)	77.2(76.3)	***93.8(95.5)***	*86.2(88.8)*	74.8(68.9)	82.7(70.6)	71.7(64.6)	76.9(63)	85.7(84.5)	86.3(83.6)
11035	88	81.9	85.1(84.6)	77(75.7)	***93.6(95.5)***	*86(88.8)*	74.2(67.2)	82.1(68.9)	71.2(63)	76.4(61.5)	85.7(84.5)	85.7(81.8)
11036	89.6	81.6	85.3(84.6)	77(75.9)	**96.2(95.6)**	*85.1(87.8)*	64(51.7)	79.8(79.3)	79.4(72.3)	85.6(73.8)	***92.1(92.7)***	83.7(81.9)
11038	87.9	81.5	85.3(85)	77(76.3)	***94.5(96)***	*86.3(88.4)*	74.8(68.9)	82.7(70.6)	60.4(56)	65.7(54.6)	85.7(84.5)	86.3(83.6)
11039	88.1	82.1	85.2(84.8)	77(75.9)	***93.6(95.5)***	*86.1(89.1)*	74.8(68.9)	82.7(70.6)	71.7(64.6)	76.9(63)	86(85.4)	86(82.7)
11045	88.1	82	85.1(84.8)	77(75.7)	***93.5(95.2)***	*86.1(88.8)*	74.8(68.9)	82.7(70.6)	71.7(64.6)	76.9(63)	86(85.4)	86(82.7)
ES90	**97.7**	83.7	**97.9(96.6)**	82.2(80.7)	**99.5(99.2)**	*85.4(88)*	79.4(75.8)	81.6(70.6)	**94.7(92)**	81.5(72.3)	**99(99)**	84(82.8)
K2	**99.6**	83	**99.3(98.5)**	82.2(81.4)	**99.8(99.6)**	*85.4(87.8)*	**100(100)**	64.9(51.7)	**100(100)**	82.5(76.9)	***99.6(100)***	84(83.7)
1123	**95.3**	84	**98.5(97.2)**	82.2(80.7)	***97.7(98.4)***	*85.7(88.3)*	64(51.7)	79.8(79.3)	80.5(73.8)	86.6(75.3)	***93(94.5)***	84.6(83.7)
11044	**99.5**	83	**99.1(97.9)**	82(80.9)	**99.7(99.6)**	*85.4(87.8)*	**100(100)**	64.9(51.7)	**100(100)**	82.5(76.9)	**99.6(99)**	84.6(84.6)
11051	**99.5**	83	**99.1(97.9)**	82(80.9)	**99.7(99.6)**	*85.4(87.8)*	**100(100)**	64.9(51.7)	**100(100)**	82.5(76.9)	**99.6(99)**	84.6(84.6)
9106	**93.3**	83.5	**92.5(90.3)**	80.9(78.3)	**97.4(96.9)**	*85.5(87.8)*	65.1(51.7)	81.6(79.3)	80.5(72.3)	87.1(73.8)	**96.3(95.4)**	84.3(83.7)
9137-5	**93.2**	83.3	**92.9(90.5)**	81.1(79.1)	**97.8(97.6)**	*85.3(88.1)*	65.1(51.7)	81.6(77.5)	80(70.7)	84.6(72.3)	***91.8(91.8)***	84(82.8)
9138	**93.2**	83.3	**92.9(90.5)**	81.1(79.1)	**97.8(97.6)**	*85.3(88.1)*	65.1(51.7)	81.6(77.5)	80(70.7)	84.6(72.3)	***91.8(91.8)***	84(82.8)
1038	**93.3**	83.4	**93(91.2)**	81.5(79.8)	**97.7(97.2)**	*85.2(88.3)*	64(51.7)	79.8(79.3)	80(73.8)	86.1(75.3)	**92.7(93.6)**	84.3(82.8)
1043	**90.7**	85.4	**91.4(90.5)**	83.3(81.7)	***93.1(93.4)***	*87.9(90.4)*	64(50)	80.4(77.5)	81(73.8)	87.1(75.3)	***93(94.5)***	84.6(83.7)
1110	**93.3**	83.4	**93(91.1)**	81.4(79.6)	**97.6(97.2)**	*85.2(88.3)*	64(51.7)	79.8(79.3)	80(73.8)	86.1(75.3)	***92.7(93.6)***	84.3(82.8)
1115	**93.3**	83.3	**93.1(91.4)**	81.3(79.6)	**97.5(97.1)**	*85.1(88.1)*	64(51.7)	79.8(79.3)	80(73.8)	86.1(75.3)	***92.7(93.6)***	84.3(82.8)
1116	**93.2**	83.3	**92.9(90.9)**	81.3(79.4)	**97.6(97.2)**	*85.2(88.3)*	64(51.7)	79.8(79.3)	80(73.8)	86.1(75.3)	***92.7(93.6)***	84.3(82.8)

Sequences with $\geq$90% identity are indicated in bold letters; Identity rates of amino acid sequences within the parenthesis; Synonymous nucleotide changes are indicated in italics.

Table S3. IBV reference strains used in this study.

IBV strains	Years of isolation	Pathogenesis	Country of origin	Accession numbers
M41	Vaccine	Respiratory	USA	GQ504725(S1-E)[a]
H120	Vaccine	Respiratory	Netherlands	GU393335(S1-E)
H52	Vaccine	Respiratory	Netherlands	EU817497(S1-E)
Arkansas DPI	1981	Respiratory	USA	GQ504720 (S1-E)
Conn46 1972	1972	NA[b]	USA	FJ904717 (S1-E)
Gray	1960	Nephropathogenic	USA	GU393334 (S1-E)
Iowa	1956	NA	USA	GU393337 (S1-E)
Beaudette	1937	Respiratory	USA	NC_001451(S1-E)
Georgia	1998	NA	USA	GQ504722(S1-E)
JMK	1964	NA	USA	GU393338(S1-E)
Holte	1954	Nephropathogenic	USA	GU393336(S1-E)
Delaware 072	1992	NA	USA	GU393332(S1-E)
Cal99	1999	NA	USA	AY514485(S1-E)
Vic S	Vaccine	Nephropathogenic	Australia	DQ490221(S1-E)
CU-T2	1995	Respiratory variant	USA	AIU49858(S1-E)
IBV-EP3	NA	NA	Singapore	DQ001338 (S1-E)
IBV-p65	NA	NA	Singapore	DQ001339(S1-E)
TW2575/98	1998	Nephropathogenic	Taiwan	DQ646405(S1-E)
TW2296/95	1995	Nephropathogenic	Taiwan	DQ646404 (S1-E)
A2	1996	Nephropathogenic	China	EU526388(S1-E)
LX4	1999	Nephropathogenic	China	AY338732(S1-E)
ZJ971	1997	Proventriculus	China	EU714028(S1-E)
BJ	1997	Nephropathogenic	China	AY319651(S1-E)
SAIBK	NA	Nephropathogenic	China	DQ288927(S1-E)
GX-YL5	2005	Nephropathogenic	China	HQ848267(S1-E)
KB8523	1988	Nephropathogenic	Japan	M21515 (S1-E)
Peafowl/GD/KQ6/2003	2003	NA	China	AY641576(S1-E)
Partridge/GD/S14/2003	2003	NA	China	AY646283(S1-E)
Turkey coronavirus	2000	NA	Canada	NC_010800(S1-E)
LDT3	2003	Nephropathogenic	China	AY702975(S1-E)
ITA/90254/2005	2005	NA	Western Africa	FN430414(S1-E)
CK/CH/LJL/04I	2004	NA	China	DQ167144(S1)<br>EF602452(S2-E)
CK/CH/LLN/98I	1998	NA	China	DQ167145(S1)<br>EF602451(S2, 3b, E)
CK/CH/LSD/031	2003	NA	China	DQ167148(S1)<br>EF602457(S2-E)
CK/CH/LTJ/95I	1995	NA	China	DQ167151(S1)<br>EF602448(S2-E)
QX	1997	Proventriculus	China	AF193423(S1)
Ma5	Vaccine	Nephropathogenic	USA	AY561713(S1)
4/91	Vaccine	Respiratory	UK	AF093794(S1)
Italy-02	1999	Respiratory	Italy	AJ457137(S1)
THA90151	2008	Respiratory	Thailand	GQ503617(S1)
K281-01	2001	respiratory	Korea	AY257062(S1)

Table S3. *Cont.*

IBV strains	Years of isolation	Pathogenesis	Country of origin	Accession numbers
K210-02	2002	Respiratory	Korea	AY257068(S1)
K083-98	1998	Nephropathogenic	Korea	FJ807936(S1)
K748-01	2001	Nephropathogenic	Korea	AY790358(S1)
K154-05	2005	Nephropathogenic	Korea	FJ807922(S1)
K283-04	2004	Nephropathogenic	Korea	FJ807923(S1)
K40-09	2009	Nephropathogenic	Korea	HM486957(S1)
K245-10	2010	Nephropathogenic	Korea	JF804686(S1)
K308-09	2009	Nephropathogenic	Korea	JF804689(S1)
K26-10	2010	Nephropathogenic	Korea	JF804678(S1)

[a] Sequences of the gene fragments used within the parenthesis. [b] NA: data not available.
